# Corepressor metastasis-associated protein 3 modulates epithelial-to-mesenchymal transition and metastasis

**DOI:** 10.1186/s40880-017-0193-8

**Published:** 2017-03-09

**Authors:** Liang Du, Zhifeng Ning, Hao Zhang, Fuxing Liu

**Affiliations:** 1 Cancer Research Center, Shantou University Medical College, Shantou, 515031 Guangdong P. R. China; 2 Basic Medicine College, Hubei University of Science and Technology, Xianning, 437100 Hubei P. R. China; 3 Department of Biotherapy, Affiliated Cancer Hospital of Shantou University Medical College, Shantou, 515031 Guangdong P. R. China

**Keywords:** Metastasis associated proteins, Coregulator, NuRD complex, Master regulator

## Abstract

Worldwide, metastasis is the leading cause of more than 90% of cancer-related deaths. Currently, no specific therapies effectively impede metastasis. Metastatic processes are controlled by complex regulatory networks and transcriptional hierarchy. Corepressor metastasis-associated protein 3 (MTA3) has been confirmed as a novel component of nucleosome remodeling and histone deacetylation (NuRD). Increasing evidence supports the theory that, in the recruitment of transcription factors, coregulators function as master regulators rather than passive passengers. As a master regulator, MTA3 governs the target selection for NuRD and functions as a transcriptional repressor. MTA3 dysregulation is associated with tumor progression, invasion, and metastasis in various cancers. MTA3 is also a key regulator of E-cadherin expression and epithelial-to-mesenchymal transition. Elucidating the functions of MTA3 might help to find additional therapeutic approaches for targeting components of NuRD.

## Background

Cancer begins as a local disease and progresses to metastatic diseases in other organs. The most devastating cancer process is metastasis, which accounts for more than 90% of cancer-related deaths worldwide [1]. Metastasis requires malignant primary tumor cells to penetrate the walls of lymphatic and/or blood vessels, circulate through the blood or lymph stream to distant organs, and colonize there to seed micrometastases. These micrometastases dedifferentiate through aberrant activation of epithelial-to-mesenchymal transition (EMT) to form a metastatic tumor [2–4]. EMT strongly enhances cancer cell motility and dissemination by dictating the interactions of cancer cells with the extracellular matrix (ECM) and neighboring stromal cells. EMT involves in the dysregulation of cell adhesion molecules (CAMs) such as integrins, immunoglobulin superfamily, and cadherins, all of which are implicated in metastasis [5–7]. Thus, activation of EMT is important for cancer cell dissemination and metastasis. EMT is a highly conserved cellular process that transforms polarized, immotile epithelial cells to migratory mesenchymal cells with stem cell-like properties. It is orchestrated by a group of transcription factors such as Snail (or SNAI1), Slug, Twist, and Zinc finger E-box-binding homeobox (ZEB) families [8, 9]. Metastasis-associated protein 3 (MTA3) has been proved as a novel component of the nucleosome remodeling and histone deacetylation (NuRD) transcriptional repression complex. As a transcriptional corepressor, MTA3 directly or indirectly regulates the activity of EMT-associated genes such as Snail and E-cadherin. A decrease of MTA3 expression leads to the up-regulation of Snail and triggers the process of EMT by repressing E-cadherin, thereby causing a loss of cell-to-cell adhesion and promoting cancer invasion and metastasis. Underexpression of MTA3 has been observed in a diverse array of human tumors [10–16]. MTA3, as a master regulator, may regulate the EMT-relevant metastasis by modulating the expression of the crucial proteins Snail and E-cadherin.

## Many roads to metastasis: EMT and beyond

**Fig. 1:**
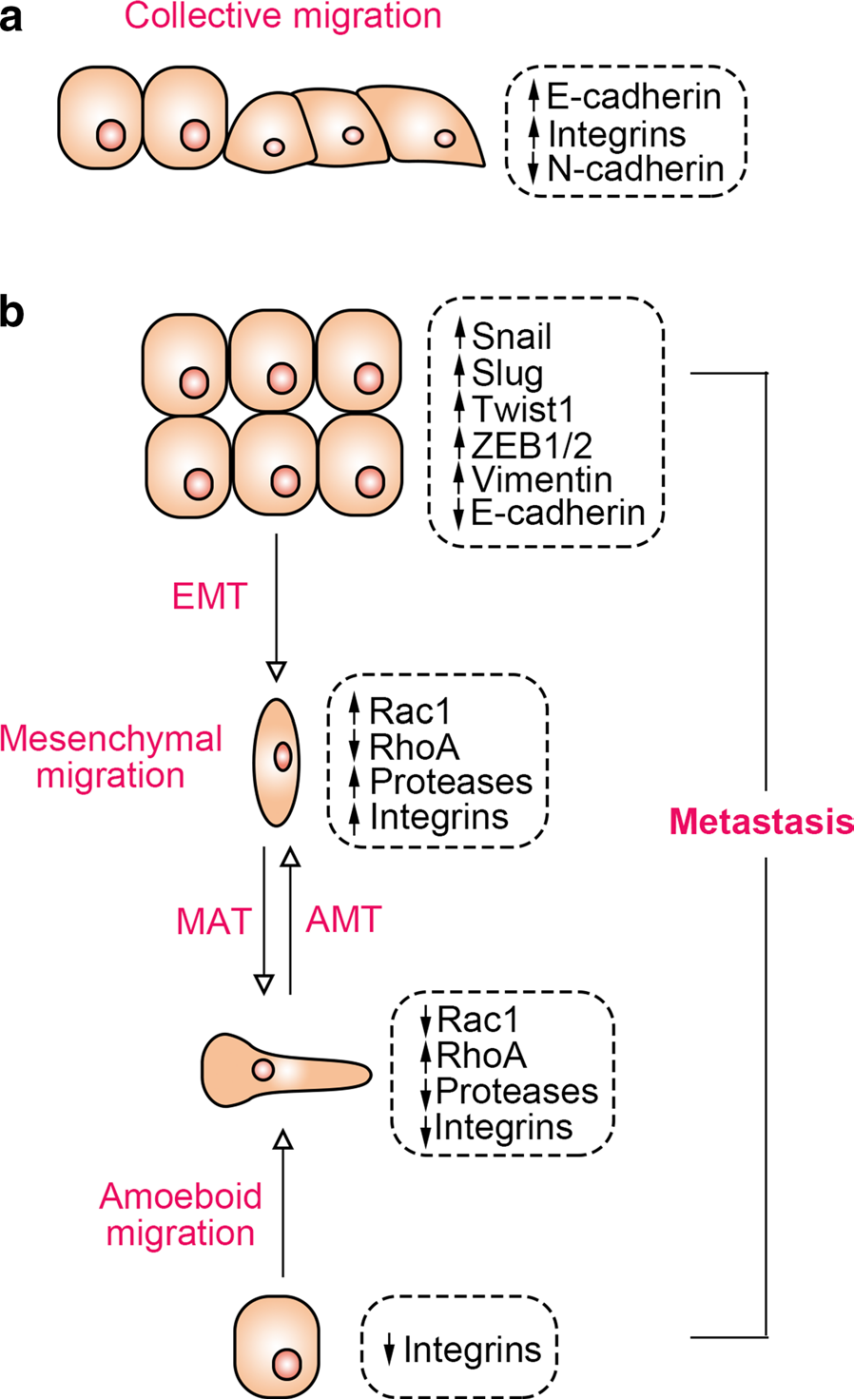
Models and transitions of tumor cell metastasis. **a** Migration of whole groups of cancer cells; **b** migration of individual cancer cell. *EMT* epithelial-mesenchymal transition, *MAT* mesenchymal-amoeboid transition, *AMT* amoeboid-mesenchymal transition, *ZEB1/2* zinc finger E-box-binding homeobox 1 and 2

Cancer metastasis is an intricate multistep process involving the detachment of cancer cells from the primary tumor, the penetration through adjacent tissue to the vasculature, and location on the distant organs where they survive and proliferate and therefore generate metastatic tumor [17–20]. Migration of cancer cells is initiated by their detachment from the ECM, then the genes that are necessary for differentiation, proliferation, and apoptosis are activated. To initiate migration, the metastatic cancer cells must undergo EMT by changing their cellular characteristics and down-regulating the expression of receptors involved in cell-to-cell adhesion. Then the cell motility is increased by down-regulating cell adhesion molecules, degrading cell-to-cell junctions, and activating proteases, finally releasing cancer cells from the ECM [9, 21, 22]. The epigenetic pattern that promotes such metastatic changes varies for different types of cancer cells, and each pattern has its unique clinical significance. The migration of cancer cells generally occurs via two different types of movement: (1) single-cell migration, involving amoeboid and mesenchymal movement, and (2) collective migration, involving the movement of cells in the form of sheets, strands, clusters, or ducts rather than individually (Fig. 1). However, two types of migrations interact intensively during cancer metastasis.

### Single-cell invasion or individual-cell migration

Single-cell tumor invasion or migration is characterized by low association in the migration pattern between cells, that is, when released from ECM, a cell has no interaction with its neighboring cells [23]. Single-cell migration occurs by amoeboid or mesenchymal movement [24–26].

#### Amoeboid cell migration

The amoeboid mechanism is the most primitive and efficient mode of single-cell migration from tumors such as lobular breast cancer [27], epithelial prostate cancer [28], leukemia [29], and melanoma [30]. This type of invasion is characterized by absent or minimal focal adhesion due to weak interaction between cells and the substratum matrix as well as absent or minimal proteolysis at the site of cell–matrix interactions, since the ECM destroying proteolytic enzymes are not expressed [25, 31–33]. All these properties determine that amoeboid cells have characteristic fast deformability and the ability to penetrate in squeezed form through small spaces of ECM [26, 34, 35]. The cell migration and relocation is accomplished through “bleb-like” pseudopodial protrusions of the cell membrane developed by alternate cycles of expansion and contraction of the cell body. These pseudopodial protrusions through their chemoreceptors sense the microenvironment and help the cells to bypass various obstacles and find the most suitable route to squeeze through narrow gaps in the ECM. Changes in the cell shape during amoeboid movement are generated by the actin cytoskeleton which is controlled by a group of molecules, including small GTPase, Rac, RhoA, and its effector ROCK kinase, that are required to reorganize the actin cytoskeleton during cell migration [24–26, 32, 36].

#### Mesenchymal cell migration

Compared with the amoeboid cell migration, mesenchymal cell migration is accomplished by more complex processes involving larger numbers of biomolecular interactions; this invasion is characterized by a spindle-shaped elongated cell body with long protrusions. Mesenchymal cell invasion has been detected during the development of breast and prostate cancers, lung carcinoma, melanoma, fibrosarcoma, glioblastoma, and many other cancers [26, 37]. During this type of migration, malignant cells gain an elongated spindle shape which resembles fibroblasts by losing their epithelial polarity; thus, this type of migration is also called “fibroblast-like” migration [24, 26, 38, 39].

The mesenchymal migration of cancer cells occurs through multiple sequential steps: (1) on one of the cell poles, the contractions of the actin cytoskeleton produces a protrusion called a lamellipodia or a filopodia under the control of small GTPases, Rac1, Cdc42, and β1 family integrins; (2) focal adhesion is generated at the site of contact between the cell and ECM involving β1 and β3 integrins; (3) assembly of focal contacts occurs due to integrin-mediated interactions and activation of matrix metalloproteinases, cathepsins, and serine and threonine proteolytic enzymes at the cell–matrix interface destructing and reorganizing the surrounding ECM; (4) myosin II-mediated change in the actin cytoskeleton polarization leads to the cell body contractions; and (5) the newly formed defects in the matrix structure pull the trailing edge toward the direction of movement [24, 26, 32, 40, 41].

### Collective cell migration

Collective cell migration is pivotal in tissue remodeling, wound healing, tissue renewal in adults, and cancer metastasis. In contrast to single-cell migration, the cells remain in constant intercellular communication during collective cell migration for the coordination of movement. Collective cell migration has been observed in the development and progression of breast cancer [42, 43], endometrial cancer [44], colorectal cancer [45, 46], melanoma [47], and oral squamous cell carcinomas [48].

Collective cell migration can be observed in two-dimensional (2D) sheet migration across a tissue surface or in multicellular strands or groups moving through a three-dimensional (3D) tissue scaffold. 2D sheets move as monolayers across tissues or along tissue clefts to form a single-layer epithelium or, after subsequent proliferation and thickening, form a multilayer epithelium. The multicellular 3D strands have a distinct basal and lateral polarization constituting an inner lumen, and therefore have a tubular structure, for instance, in morphogenic duct and gland formation or vascular sprouting during angiogenesis, or they can move as a poorly organized strand-like mass, such as in invasive cancer. Alternatively, isolated groups or clusters of cells can migrate through tissue if they detach from their origins; for example, metastatic cancer cell clusters penetrate the stromal tissues [44, 49, 50].

The collective cell migration involves two types of cells: the “leader” cells forming the leading edge that generates adhesion and traction towards the tissue substrate and the “follower” cells that are located behind them. Leader cells acquire the mesenchymal phenotype with less distinct ordering and structural organization; however, toward the trailing edge the follower cells display an apical formation of tight junctions before being deposited and tend to form more tightly packed rosette-like tubular cluster of cells. These leader cells direct multicellular aggregates through degradation of the ECM components at the invasion front, and the cells of the inner and trailing edge are dragged forward. Since they play the dominant roles in the movement of cell collectives, the leader cells are of great significance in relation to EMT. Collective migration is used by epithelial cancer cells as well as by mesenchymal cancer cells [47, 51–53].

### EMT

EMT is a type of migrating movement that belongs to collective-individual transition. EMT is considered one of the crucial initiative steps for cancer cell metastasis, which enhances the migratory capacity of cancer cells by promoting epithelial cells to lose their polarity and intercellular adhesion to acquire mesenchymal features [3]. During the EMT process, epithelial cells acquire a fibroblastic motile phenotype by losing their cell–cell adhesion properties and apical-basal polarity. In solid tumors, migrating tumor cells are produced by EMT. In turn, migrating tumor cells that have undergone EMT reach and reside in metastatic organs and are able to form tumor multicellular complexes by regaining an epithelial phenotype called mesenchymal-to-epithelial transition (MET) [54, 55]. Hallmarks of EMT are the down-regulation of E-cadherin and up-regulation of vimentin, which are tightly controlled by multiple signaling cascades. Transforming growth factor-β (TGF-β) plays most prominent role in promoting the conversion of epithelial to mesenchymal characteristics by transcriptional and post-transcriptional regulation of a distinct set of transcription factors [56–61]. A variety of transcriptional factors such as the zinc finger Snail homologues (Snail1 and Snail2/Slug) and different basic helix-loop-helix factors (Twist, ZEB1, and ZEB2), which are capable of triggering cellular reprogramming, have been demonstrated to promote EMT through the coordinated modulation of EMT-related genes [62–66].

Multiple extracellular stimuli, including epidermal growth factor (EGF), hepatocyte growth factor (HGF), Notch, Wnt, TGF-β, and platelet-derived growth factor (PDGF), orchestrate the EMT-related process by integrated networks of signal transduction pathways and transcription factors. Transcription factors such as Snail, Twist, ZEB, and histone deacetylase (HDAC), which are capable of triggering cellular reprogramming, are the key strength of EMT. Since the expression of these EMT-relevant transcription factors is tightly orchestrated by transcriptional hierarchy, coregulators play a master role in the expression of transcription factors that subsequently regulate EMT and metastasis [67–69].

MicroRNAs (miRNAs) also crucially regulate EMT and have been found to be dysregulated in diverse array of human cancers [70]. miRNAs play an important role in the control of cell growth, differentiation, maturation, and apoptosis, which are critical for the development and progression of cancer [71]. miRNAs are also involved in the regulation of multiple signaling pathways in EMT [71, 72]. miR-21, an identified “oncomiR,” has been implicated in the promotion of EMT [73]. Inhibition of miR-21 was sufficient to inhibit EMT and stemness [74]. miR-506 in EMT inhibition has also been demonstrated in several other cancer types [71, 75, 76], indicating that miR-506 functions as a tumor suppressor in a wide spectrum of cancers. Other well-known miRNAs regulating EMT are miR-101, miR-200c, and miR-141 [71].

## Master coregulators: metastasis promoters and suppressors

Transcriptional coregulators are a large family of proteins that either activate (coactivator) or repress (corepressor) the transcription of specific genes by interacting with transcription factors. These proteins can be recruited to the enhancer or promoter regions of target genes through interaction with transcription factors to mediate their transcriptional potency, even though they do not have intrinsic DNA-binding capacity. Transcription coregulators regulate the expression of a gene either by modifying chromatin structure through covalent modification of histones or modifying chromatin conformation in an ATP-dependent manner. In contrast to recruitment by transcription factors, coregulators are recognized as master regulators that coordinately control groups of genes at the transcriptional level. Increasing evidence has shown that coregulators have more versatile functions in elongation, splicing, and further translation.

Coregulators embrace the efficacy and selectivity for sub-reactions of transcription and critically influence tissue-selective gene functions, including maintenance of cell proliferation, differentiation, adhesion, migration, and apoptosis. Since each tissue has a specific expression profile and concentration of coregulators for maintaining its normal homeostasis, any alteration in cellular concentration of coregulators may lead to functional dysregulation of molecular machinery and genetic instability of the cell or specific tissue, which cause pathologic complications such as cancers. In many cancers, coregulators are mis-expressed and are hijacked by these cancer cells to modulate their sustained proliferation and metastasis.

Emerging evidence indicates that coregulators play a regulatory role in EMT and cancer metastasis. In this regard, coactivators such as steroid receptor coactivators (SRCs, i.e., SRC-1, SRC-2, and SRC-3), proline, glutamate, and leucine rich protein 1 (PELP1) [77], peroxisome proliferator-activated receptor γ coactivator-1 (PGC-1) [78], and Yes-associated protein (YAP) [79], as well as corepressors including metastasis-associated protein family (i.e., MTA1, MTA2, and MTA3) [80], nuclear receptor corepressor (N-CoR) [81, 82], silencing mediator of retinoic acid and thyroid hormone receptor (SMRT) [81], switch-independent 3A (Sin3A) [83], C-terminal binding protein (CtBP) [84], and HDAC3 [85], have been reported to regulate tumor cell invasion and metastasis.

Many coregulators are capable of mediating both tumor growth and metastasis. The steroid receptor coactivator (SRC) family is the major coactivator for nuclear receptor (NR)-dependent transcription [86]. The SRC family consists of three members: SRC-1, SRC-2, and SRC-3. In cancers, both SRC-2 and SRC-3 not only promote tumor growth but also mediate metastasis [87, 88]. SRC-3 also plays a pivotal role in constitutive androstane receptor (CAR) activation and promotes proliferation and drug metabolism in the liver [89]. Interestingly, data derived from studies on breast cancer showed that SRC-1, the first discovered and cloned coactivator, is exclusively responsible for promoting metastasis without accelerating tumor growth [90]. SRC-1 plays a vital role in cancer cell invasion through multiple mechanisms by modulating twist, polyoma enhancer activator 3 (PEA3), Snail, and Smad interacting protein 1 (SIP1) [91, 92]. Thus, it is possible that SCR-1 may fall into category of proteins that are defined as potent metastasis suppressors.

## MTA proteins: regulators of metastasis

**Fig. 2:**
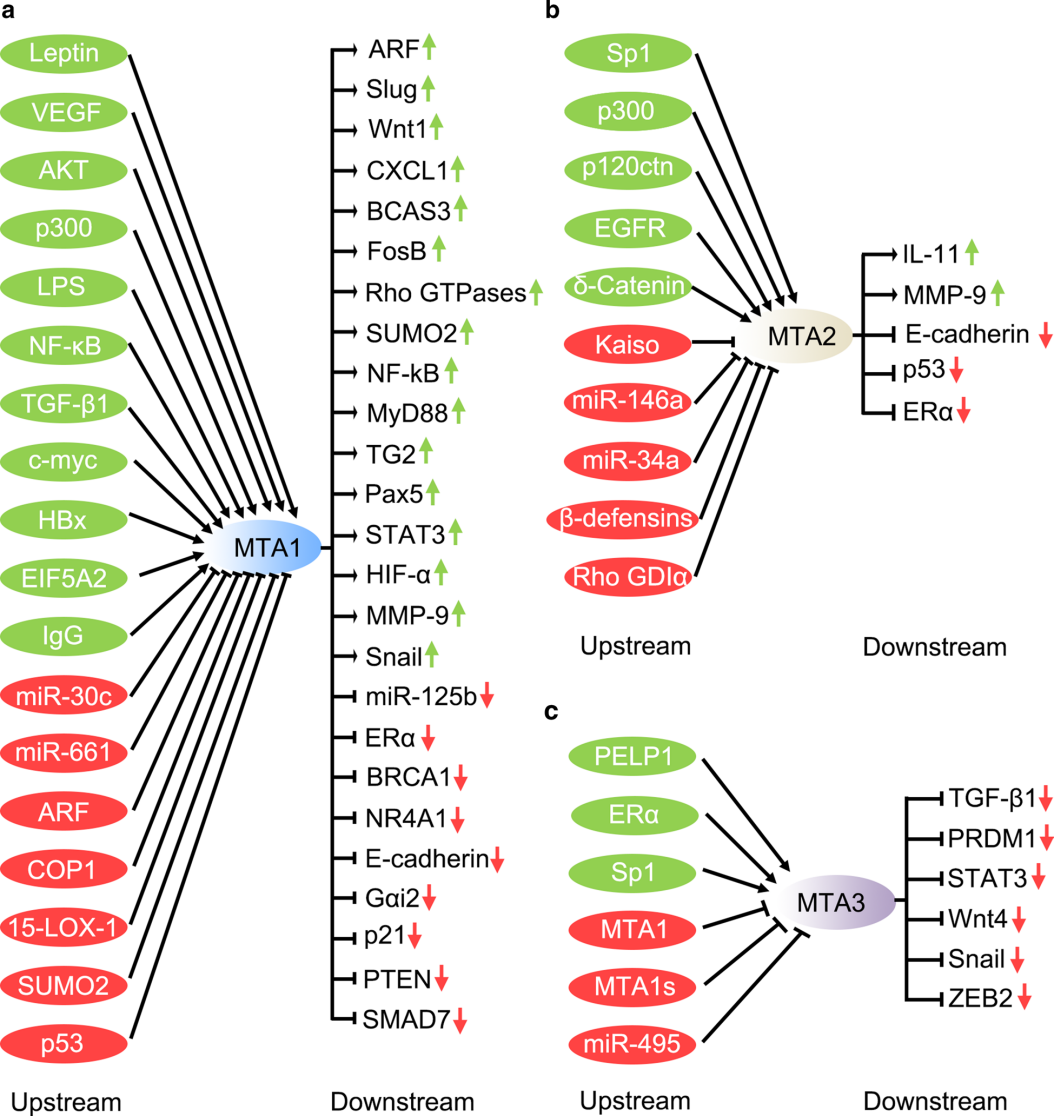
Upstream regulators and downstream effectors of metastasis-associated proteins (MTAs) in human cancers. Upstream regulators that directly or indirectly up-regulate or down-regulate MTA1 (**a**), MTA2 (**b**), and MTA3 (**c**) are listed on the *left side*, whereas downstream effectors that are directly or indirectly regulated by MTAs are listed on the corresponding *right side*. *VEGF* vascular endothelial growth factor, *AKT* protein kinase, *LPS* lipopolysaccharide, *NF*-*κB* nuclear factor kappa-light-chain-enhancer of activated B cells, *TGF*-*β1* transforming growth factor beta 1, *HBx* hepatitis B viral protein, *EIF5A2* eukaryotic translation initiation factor 5A-2, *ARF* alternative reading frame, *COP1* coat protein 1, *15*-*LOX*-*1* 15-lipoxygenase-1, *SUMO2* small ubiquitin-related modifier 2, *CXCL1* chemokine (C-X-C motif) ligand 1, *BCAS3* breast carcinoma-amplified sequence 3, *TG2* transglutaminase 2, *Pax*-*5* paired box protein 5, *STAT3* signal transducer and activator of transcription 3, *HIF*-*α* hypoxia-inducible factor-α, *MMP*-*9* matrix metallopeptidase 9, *miR*-*125b* microRNA-125b, *ERα* estrogen receptor alpha, *BRCA1* breast cancer 1, *NR4A1* nuclear receptor subfamily 4 group A member 1, *Gαi2* Gi alpha subunit 2, *PTEN* phosphatase and tensin homolog, *SMAD7* mothers against decapentaplegic homolog 7, *Sp1* specificity protein 1, *p120ctn* p120 catenin, *EGFR* epidermal growth factor receptor, *GDIα* GDP dissociation inhibitor alpha, *hBD*-*3* human β-defensin 3, *IL*-*11* interleukin-11, *PELP1* proline, glutamic acid, leucine-rich protein 1, *MTA1* *s* metastasis-associated protein 1s, *PRMD1* PR domain containing 1, with ZNF domain, *ZEB2* zinc finger E-box-binding homeobox 2

Metastasis-associated protein (MTA) family is an emerging family of novel transcriptional coregulators that are specifically relevant to metastasis regulation, which comprises six members—MTA1, MTA1s, MTA-ZG29p, MTA2, MTA3, and MTA3L—that are separately encoded. MTA family members generally form independent NuRD complexes that repress transcription by recruiting histone deacetylases on different target genes. MTAs are the key components of the NuRD complex, which show a crucial role in cancer cell invasion and metastasis, associating with a variety of cancer-related factors such as Snail, E-cadherin, and signal transducer and activator of transcriptions (STATs) [80]. Furthermore, MTAs are also regulated by various factors [10, 80, 93, 94] (Fig. 2). The absence of estrogen receptor or MTA3 leads to aberrant up-regulation of Snail, resulting in loss of E-cadherin expression. MTA3 protein is required in normal development for sustaining the controlled cell growth and homeostasis and, in cancers, to combat the spread of cancers through EMT and metastasis [95]. Interestingly, MTA protein family members have been identified as transcription corepressors; however, they may form functionally specialized NuRD complex and then display varied roles in cancer initiation, progression, and metastasis. MTA1 is the founder of the MTA family and is found to be overexpressed in breast cancer [80], causing increased EMT, migration, and metastasis; however, there are remarkable differences among MTA3, MTA1, and MTA 2.

MTA3 was identified as a component of Mi2/NuRD complex and transcriptional corepressor, which is dependent on estrogen and negatively regulate gene expression in breast cancer cells [21]. In response to estrogen, a distinct MTA3-Mi2/NuRD transcriptional corepressor complex is formed, which contains histone deacetylase and has ATP-dependent chromatin remodeling functions [10]. This complex modulates the expression of E-cadherin by inhibiting expression of Snail [10], which further blocks EMT. Since MTA3 is a transcriptional target of estrogen receptor-α (ERα), the function of MTA3 is linked to the ERα pathway. In the presence of ligand, ERα directly binds to the MTA3 promoter at the half-estrogen response element (ERE)/Sp1-binding site and stimulates MTA3 transcription [96, 97]. Since both MTA1 and MTA1 s negatively regulate ERα function and *MTA3* is an estrogen-regulated gene, any probable up-regulation of MTA1 or MTA1 s may lead to decrease of the expression of MTA3. Any regulated reduction in the level of MTA3 will lead to up-regulation of Snail, enhancement of EMT, and metastasis of breast cancer. MTA3 influences the Wnt signaling and directly represses Wnt4 transcription [11], which countermands Snail activation induced by Wnt [98].

## Association of MTA3 with EMT and metastasis in cancers

**Fig. 3:**
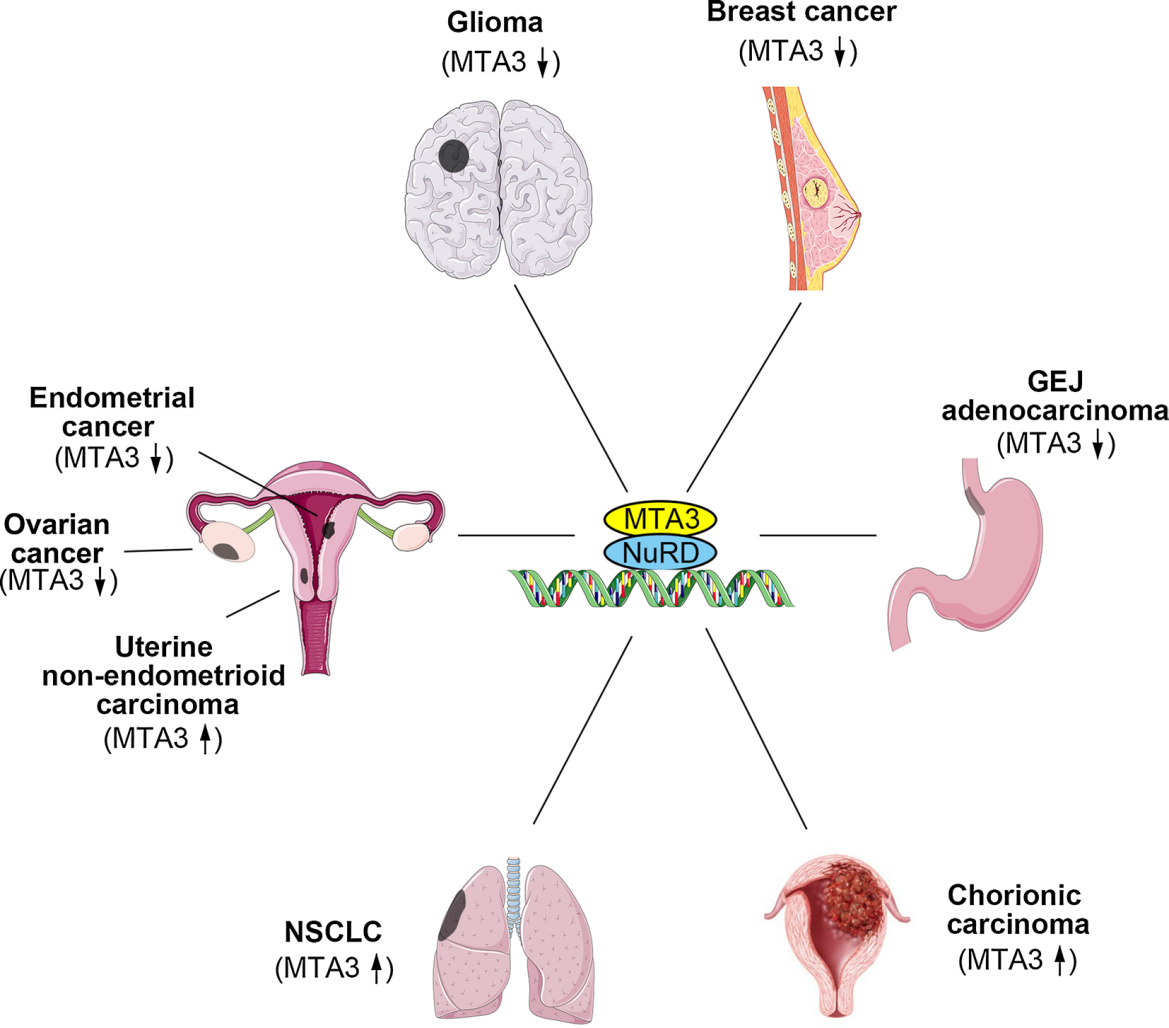
Aberrant expression of MTA3 in a host of human tumor types. *NSCLC* non-small cell lung cancer, *GEJ* gastroesophageal junction, *NuRD* nucleosome remodeling and histone deacetylation

Although MTA3 is involved in multiple cellular activities in physiological and pathologic processes, MTA3 has been extensively studied for its regulation and association with EMT and metastasis in cancer (Fig. 3).

### Underexpression of MTA3 in cancers

MTA3 was first found to be down-regulated in cancers and repress EMT and invasion.

#### Breast cancer

MTA3 was originally identified as a corepressor that inhibits breast cancer cell EMT, invasion, and metastasis [10], and its protein expression is gradually decreased during progression in breast cancer tissues [11]. In cultured ER-positive MCF-7 cell lines, depletion of MTA3 increases expression of Snail, which regulates EMT, and in turn reduces E-cadherin expression and improves invasive growth [10]. Because MTA3 is a cell type-specific component of the NuRD complex, and MTA3 expression depends on estrogen action, MTA3 regulates EMT and cancer metastasis of breast cancer via the ER-MTA3/NuRD/Snail/E-cadherin pathway. Using mouse mammary tumor virus polyoma virus middle T (MMTV-PyV-mT) transgenic mouse model, MTA3 expression was compared with that of MTA1 and MTA2 in normal duct, premalignant lesions, invasive carcinoma, and metastatic tumors [11]. The results showed that MTA3 protein expression had a positive association with that of E-cadherin and cytoplasmic β-catenin and that MTA3 protein expression was progressively reduced during breast cancer progression [11]. These results proved that MTA3 exhibits a critical role in EMT and cancer metastasis.

#### Gastroesophageal junction adenocarcinoma

Gastroesophageal junction (GEJ) adenocarcinoma is a malignancy that shows frequent metastasis. In GEJ adenocarcinoma, the components of the MTA3 pathway were proved to be of prognostic significance [15]. Down-regulation of MTA3 mRNA and protein was detected in tumor tissues compared with non-tumor tissues; MTA3 levels were significantly lower in tumor cell lines with stronger metastatic potential compared with tumor cell lines with less metastatic potential [15]. It was also observed that the patients with low MTA3 expression had poor prognosis [15]. Furthermore, the malignant properties were found to be strongly associated with the abnormal expression of MTA3, Snail, and E-cadherin [15], suggesting that MTA3 regulates EMT and promotes metastasis via repressing Snail expression. These data reveal that MTA3 can serve as an independent prognostic factor for patients with GEJ adenocarcinoma.

#### Glioma, ovarian cancer, and endometrial cancer

MTA3 is underexpressed in glioma [99], ovarian cancer [100], and endometrial cancer [101]. MTA3 expression was reported to be associated with differentiation [13], cancer progression [14], and overall survival rates [14, 99]. Given that the malignancies such as glioma, ovarian cancer, and endometrial cancer have nature of invasive and migration, further studies are required to elucidate how MTA3 regulates the EMT and metastasis in these cancers.

### Up-regulation of MTA3 in cancers

Besides down-regulation, MTA3 was also found to be up-regulated in cancers [12, 14, 102]. Compared with MTA1 and MTA2, MTA3 may have more complex functions in cancer progression.

#### Non-small cell lung cancer

Non-small cell lung cancer (NSCLC) represents approximately 85% of lung cancers; approximately 40% of NSCLC patients have poor prognosis due to cancer cell invasion [103]. It has been observed that MTA3 was overexpressed in NSCLC tissue, which can serve as a risk factor for lymph node metastasis [102]. Furthermore, MTA3 was found to be a target of miR-495, which inhibited proliferation and migration in lung cancer cells [102]. These findings suggest that miR-495 could be of great clinical importance in targeting MTA3 for regulating lung cancer growth and migration.

Metastasis and therapeutic resistance have been demonstrated to be the major causes of the failure of cancer treatment [104]. A body of evidence has identified EMT as a key step for facilitating cancer metastasis and radioresistance [105]. For example, liver kinase B1 (LKB1)-salt-inducible kinase 1 (SIK1) signaling has been shown to suppress EMT [106]. In radioresistant NSCLC cells, LKB1-SIK1 signaling was attenuated; however, radiosensitivity of NSCLC cells was increased by re-expression of LKB1 [107]. Since MTA3 was involved in the regulation of EMT by miR-495 in NSCLC cells [102], it is reasonable to speculate that MTA3 may also play a role in regulation of therapeutic resistance in NSCLC.

#### Chorionic carcinoma

Human chorionic carcinoma is an aggressive and metastatic carcinoma. It is well known that MTA3 is involved in cancer cell migration by regulating cell adhesion proteins. Bruning et al. [12] recently reported high expression of MTA1 and MTA3 in the nuclei of human chorionic carcinoma cells, suggesting that high expression level of MTA proteins might facilitate trophoblast cell migration and neoangiogenesis.

#### Uterine non-endometrial cancer

Non-endometrioid carcinoma, a highly malignant form of endometrial cancer, has a poor prognosis, mostly due to its increased tendency for extra-uterine metastasis [108]. In contrast to its underexpression in endometrioid carcinomas, MTA3 was found to be overexpressed in uterine non-endometrial cancer [14]. MTA3 overexpression was positively associated with high International Federation of Gynecology and Obstetrics (FIGO) surgical stage, lymph node metastasis, and lymphovascular space invasion (LVSI) [14]. Patients with a higher MTA3 expression were more likely to have shorter progression-free, cause-specific, and overall survival compared with those with a lower MTA3 expression [14]. Interestingly, MTA3 can be considered an independent prognostic factor only for cause-specific survival [14]. These data indicated that elevated MTA3 expression might contribute to a more aggressive phenotype in non-endometrial cancer. It would be extremely intriguing and important to examine whether MTA3 expression could serve as a biomarker to differentiate endometrioid from non-endometrioid carcinomas.

### MTA3 regulates many metastasis-relevant genes

MTA3 in the NuRD complexes was found to target a set of genes that may be involved in EMT and metastasis [95]. The first notion about the molecular and biochemical functions of MTA3 was revealed by Fujita et al. [10], who reported MTA3 as an estrogen-inducible gene product that forms a distinct NuRD complex with strong transcription-repressing activity on Snai1 and then up-regulates E-cadherin, subsequently inhibiting EMT. Later, MTA3 was also found to interact with the Wnt4-containing chromatin in an HDAC-dependent manner, thus, repressing Wnt4 transcription [109]. Since a role of Wnt signaling for breast cancer metastasis has been described [1], it is reasonable to speculate that MTA3 might inhibit metastasis via suppressing Wnt signaling. Lysine-specific demethylase-1 (LSD1) is a physical integral component of the MTA3/NuRD complex in vivo; the LSD1/MTA3/NuRD complex targets TGFβ1, then inhibits the invasiveness in vitro and suppresses metastatic potential in vivo in breast cancer [110]. More recently, the MTA3/NuRD complex was reported to be physically associated with GATA-binding protein 3 (GATA3) and G9A, and the functional GATA3/G9A/NuRD (MTA3) complex can inhibit ZEB2 [93]. The authors postulated a new mechanism in MTA3-mediated control of EMT and cell invasion in breast cancer [93]. Moreover, these results suggested that dysregulation of the reciprocal feedback between GATA3/G9A/NuRD (MTA3) and ZEB2/G9A/NuRD (MTA1) may contribute to breast cancer progression.

## Summary and future insight

In summary, MTA3 is a decisive modulator for EMT and metastasis in cancers. However, our understanding of MTA3 mechanism is the tip of the iceberg, and many questions still need addressing. For instance, it remains to be determined whether MTA3 regulates other metastatic manners in addition to EMT. Besides transcriptional initiation, coregulators have been found to be involved in elongation, splicing, and further translation. However, does MTA3 have more functions than transcriptional coregulator? Current evidence shows that MTA3 acts in a HDAC-dependent manner. It is unclear whether MTA3 could function in the HDAC-independent pathway. From a translational viewpoint, the association between MTA3-mediated signaling, aggressiveness, and clinical outcomes has not been fully examined in different cancers.

Cellular activities in cancer metastasis are controlled by a hierarchy of different mechanisms. One of such important molecular mechanisms is involved in modification at transcriptional levels through NuRD-mediated chromosomal remodeling. Elucidating functions of MTA3 might provide further approaches for targeting components of NuRD for therapeutic purposes.
